# The relationship between spatial ability self-efficacy and visualization performance: psychometric evidence

**DOI:** 10.3389/fpsyg.2026.1829983

**Published:** 2026-07-22

**Authors:** Okan Kuzu, Hanife Taşdelen

**Affiliations:** 1 Faculty of Education, Kırşehir Ahi Evran University, Kırşehir, Türkiye; 2 Institute of Natural and Applied Sciences, Kırşehir Ahi Evran University, Kırşehir, Türkiye

**Keywords:** self-efficacy, spatial ability, spatial orientation, spatial relations, spatial visualization

## Abstract

**Introduction:**

This study adopts a holistic perspective on spatial ability by integrating preservice teachers’ self-efficacy perceptions with performance-based approaches. It aims to assess spatial ability self-efficacy and examine differences across selected variables, as well as the relationship between spatial self-efficacy and visualization performance.

**Methods:**

A valid and reliable measurement instrument, the Spatial Ability Self-Efficacy Perception Scale, was developed. The scale consists of 35 items across three factors—spatial visualization, spatial relations, and spatial orientation—and demonstrates high internal consistency (Cronbach’s *α* = 0.960).

**Results:**

The findings indicate that preservice teachers generally report high levels of spatial self-efficacy, with higher levels in spatial visualization and spatial orientation and moderate levels in spatial relations. While no significant gender differences were observed, spatial self-efficacy perceptions differed significantly by academic program, with higher levels among preservice teachers in Mathematics Education as well as Social Studies Education. In addition, the total spatial self-efficacy perception score showed a positive and moderate association with visualization performance; at the factor level, spatial visualization and spatial relations showed moderate associations, whereas spatial orientation showed a weak association.

**Discussion:**

Overall, the results support conceptualizing spatial ability as a multidimensional construct shaped by the interaction of cognitive performance and self-efficacy perceptions. By integrating affective and cognitive dimensions of spatial thinking, this study offers psychometric, methodological, and theoretical contributions to spatial ability research and provides implications for teacher education.

## 1 Introduction

Individuals’ ability to interpret objects, shapes, and spatial structures in their environment relies on a set of higher-order cognitive processes, including mental representation, transformation, and flexible reorganization of spatial information. Beyond the mere mental construction of a shape, the ability to consider alternative positions and orientations and to express these representations through drawing requires complex cognitive and perceptual processing. In the psychological literature, such processes are conceptualized within the framework of spatial ability, reflecting an individual’s capacity to encode, manipulate, and utilize spatial information effectively.

Within educational contexts, spatial ability has long been assessed predominantly through performance-based measurement instruments. These assessments capture the extent to which individuals can accurately and efficiently perform two- and three-dimensional spatial tasks. However, performance in spatial tasks is not solely determined by cognitive capacity; it is also closely associated with individuals’ self-efficacy perceptions regarding their ability to successfully perform such tasks. Self-efficacy perceptions influence task selection, effort expenditure, and persistence in the face of difficulties, thereby playing a critical role in shaping actual performance.

In the literature, spatial ability has been examined within the framework of various variables such as age and grade level, gender, mathematics achievement, socioeconomic context, and instructional experiences. Previous research has demonstrated that spatial skills are significantly associated with both developmental patterns and academic performance (Abay et al., 2018; Linn and Petersen, 1985; Uttal et al., 2013; Verdine et al., 2014; Atit et al., 2022). However, studies that directly investigate the relationship between individuals’ self-efficacy perceptions regarding spatial ability and their actual visualization performance remain limited. Yet, in spatial tasks, individuals’ self-efficacy perceptions about their capability to perform these tasks may be as critical as their actual level of success, particularly in terms of performance emergence and persistence. This issue becomes especially salient for preservice teachers, for whom not only accurate execution of spatial tasks but also strong self-efficacy perceptions in their ability to successfully perform such tasks can directly shape the problem-solving approaches they employ in instruction and the learning experiences they design for students. Therefore, examining the relationship between spatial ability self-efficacy and visualization performance has the potential to contribute to a more comprehensive understanding of spatial thinking within the context of teacher education.

This study aims to examine the relationship between spatial ability self-efficacy perceptions and visualization performance, rather than conceptualizing spatial ability solely as a performance-based construct. In this respect, by elucidating the interaction between individuals’ self-evaluations and objective performance, the study seeks to make original contributions to the literature on spatial thinking across the domains of measurement, teacher education, and educational practice.

## 2 Background

Spatial ability has been extensively examined across multiple disciplines, particularly within cognitive psychology and educational research. Although varying approaches exist regarding the definition, dimensions, and measurement of spatial ability, there is broad consensus that spatial ability constitutes a multidimensional cognitive construct. Within this framework, spatial ability is conceptualized as a set of cognitive processes involving the mental visualization and transformation of objects, as well as the perception of spatial relationships. Early studies on the structure of spatial ability tended to define this construct in more holistic terms, whereas subsequent research has increasingly focused on its distinct dimensions. In particular, spatial visualization, spatial orientation, and spatial relations have emerged as the most frequently emphasized core dimensions across theoretical perspectives. Together, these dimensions encompass both the static and dynamic aspects of spatial information, thereby accounting for multiple dimensions of spatial thinking.

In the literature, spatial ability has been predominantly assessed through performance-based tests, with emphasis placed on individuals’ accuracy and speed in completing spatial tasks (Morawietz et al., 2024; Yoon, 2011; Ung et al., 2016). However, recent research suggests that spatial self-efficacy should also be considered in spatial learning and task contexts, as individuals’ self-efficacy perceptions may be associated with effort, persistence, engagement, and performance-related outcomes (Arıkan and Çetin, 2024; Miola et al., 2021; Rérat and Berger, 2026).

The literature reveals a limited number of studies that jointly examine self-efficacy perceptions related to spatial ability and actual visualization performance. Particularly within samples of preservice teachers, a comprehensive investigation of the relationship between self-efficacy perceptions and performance is essential for a deeper understanding of the role of spatial thinking in teacher education. Addressing this gap, the present study systematically examines the relationship between spatial ability self-efficacy perceptions and visualization performance.

### 2.1 Spatial ability

Spatial ability refers to a set of cognitive skills related to an individual’s capacity to mentally visualize objects in space, recognize these objects from different perspectives, and mentally manipulate objects or their parts independently (Erdenechimeg and Danaa, 2023). In addition, spatial ability has been defined as a collection of mental processes involving the visual representation, comprehension, orientation, reorganization, and interpretation of spatial relationships (Tartre, 1990). This ability enables individuals to determine their own position relative to objects in space and to anticipate the trajectory of a moving object (Freina and Ott, 2014). According to Linn and Petersen (1985), spatial ability is a cognitive function that involves the structuring, transformation, and recall of information that is difficult to express through symbolic or verbal means. Similarly, Clements (1998) conceptualized spatial ability as dynamic cognitive processes through which mental representations of spatial objects, relationships, and transformations are constructed.

In daily activities, spatial ability is engaged in tasks such as object placement, navigation, and vehicle operation, while in professional domains it plays a critical role in areas including architectural design, surgical practice, and advanced mathematical problem solving (Rafi et al., 2008; Uttal et al., 2013; Wai et al., 2009).

A review of the literature indicates that there is no complete consensus among researchers regarding the structure and dimensions of spatial ability. Across studies, spatial ability has been conceptualized under different classification frameworks, and similar cognitive processes have often been described using varying conceptual labels.

Table 1 presents the historical development of theoretical approaches to the dimensions of spatial ability and highlights points of convergence and divergence among researchers. In early studies, spatial ability was conceptualized through more holistic and general structures. Within this framework, Maccoby and Jacklin (1974) examined spatial ability under two main factors: analytic and non-analytic. They defined the analytic factor as involving tasks that require complex mental operations, whereas the non-analytic factor was characterized by processes that require the mental rotation of objects. This approach is regarded as one of the first systematic distinctions indicating that spatial ability involves different cognitive processes.

**Table 1:** Conceptualizations and classification frameworks of spatial ability in the literature.

Classification category	Maccoby and Jacklin (1974)	McGee (1979)	Lohman (1979)	Pellegrino et al. (1984)	Linn and Petersen (1985)	Tartre (1990) and Clements (1998)	Contero et al. (2005), Pittalis and Christou (2010), Risma et al. (2013), and Ung et al. (2016)	Lowrie and Logan (2018)
Analytical	✓							
Non-analytical	✓							
Spatial orientation		✓	✓			✓	✓	✓
Spatial relations			✓	✓			✓	
Spatial visualization		✓	✓	✓	✓	✓	✓	✓
Spatial perception					✓			
Mental rotation					✓			✓

In subsequent years, a strong consensus emerged that spatial ability constitutes a multidimensional cognitive construct. Particularly since the late 1970s, the dimensions of spatial ability have been defined more clearly and systematically. In this context, Lohman (1979) conceptualized spatial ability as a three-factor structure consisting of spatial visualization, spatial relations, spatial orientation and proposed a foundational theoretical framework in the literature. This classification is noteworthy in that it addresses both the static and dynamic cognitive processes of spatial ability within an integrated structure.

An examination of the studies presented in Table 1 indicates that spatial visualization appears as a common dimension in almost all classifications. This finding suggests that spatial visualization is one of the core dimensions of spatial ability and is consistently acknowledged across different theoretical approaches. Similarly, spatial orientation and spatial relations became more prominent particularly in studies conducted after Lohman (1979) and have been treated as fundamental dimensions whose importance has been sustained in many investigations (Contero et al., 2005; Pittalis and Christou, 2010; Risma et al., 2013; Ung et al., 2016). In contrast, spatial perception has been considered as an independent dimension in only a limited number of studies (e.g., Linn and Petersen, 1985). Linn and Petersen (1985) defined spatial perception as the ability to accurately determine spatial relations relative to one’s own body position despite distracting stimuli, while mental rotation was conceptualized as the capacity to rapidly and accurately rotate two- and three-dimensional figures mentally. However, in subsequent studies, mental rotation has generally been conceptualized not as an independent construct but as a fundamental cognitive process embedded within the dimensions of spatial visualization and spatial relations.

Table 1 and this integrative review of the literature indicate that theoretical approaches to spatial ability largely converge around the dimensions of “spatial visualization,” “spatial relations,” and “spatial orientation” with these three dimensions emerging as the core dimensions of spatial ability. In the present study, these three dimensions are adopted as the theoretical framework of spatial ability, in line with the classifications widely used in the literature. At the psychometric level, these dimensions correspond to the three factors of the Spatial Ability Self-Efficacy Perception Scale (SASEPS). At the item-content level, each factor is represented through specific cognitive processes such as “mental integration,” “mental decomposition,” “mental rotation,” “viewpoint-based representation,” and “plan-based representation.”

### 2.2 Dimensions of spatial ability

#### 2.2.1 Spatial visualization

Spatial visualization is a cognitive ability that involves the mental construction and transformation of two- and three-dimensional objects, as well as the execution of multi-step mental operations on these objects. According to McGee (1979), spatial visualization is associated with the mental generation and transformation of two- and three-dimensional objects. Lohman (1979), in turn, defined this dimension through tasks that involve multi-stage and complex mental operations, such as paper folding. Similarly, Pellegrino et al. (1984) conceptualized spatial visualization as tasks that require accuracy and complexity rather than speed, including mental manipulations among the parts of structures and the folding and unfolding of planar models. Linn and Petersen (1985) described spatial visualization as tasks involving complex and multi-step manipulations of spatially presented information, noting that these tasks also encompass spatial perception and mental rotation processes. Tartre (1990) defined spatial visualization as tasks requiring multifaceted mental operations and emphasized that the key criterion distinguishing this dimension from spatial orientation lies in what is mentally moved. According to the researcher, when the whole object or a part of it is mentally moved or altered within a representation, the process falls within the scope of spatial visualization. Clements (1998) similarly defined spatial visualization as the ability to understand and mentally perform the imagined movements of two- and three-dimensional objects, emphasizing that this process requires the formation of a mental image and the deliberate manipulation of that image. Contero et al. (2005) likewise defined spatial visualization as the ability to create a mental image of objects and spatial forms and to operate on that image.

#### 2.2.2 Spatial relations

Spatial relations constitute a domain of cognitive skills that involves the mental processing of positional connections between two- and three-dimensional objects and the accurate transformation of these objects across different orientations. According to Lohman (1979), spatial relations are associated with the mental rotation of two- and three-dimensional objects and the construction of their various positions in the mind. Similarly, Pellegrino et al. (1984) defined spatial relations as the ability to perform mental transformation and rotation processes of an object quickly and accurately. Contero et al. (2005) explained spatial relations primarily in terms of the mental rotation of objects in a two-dimensional plane, conceptualizing this dimension as requiring the rapid and accurate mental rotation of a spatial object. More recent studies in the literature support this perspective by defining spatial relations as the ability to accurately perform mental rotations and positional transformations of spatial objects (Pittalis and Christou, 2010; Risma et al., 2013; Ung et al., 2016).

#### 2.2.3 Spatial orientation

Spatial orientation is associated with the mental reconstruction of an object’s position and orientation in space based on an individual’s viewpoint and perceptual perspective, without mentally moving the object itself. In this context, McGee (1979) explained spatial orientation in terms of viewpoint-dependent mental position changes when the object remains fixed, whereas Lohman (1979) defined the ability to mentally visualize how objects appear from different perspectives as the core feature of spatial orientation. Tartre (1990) conceptualized spatial orientation as a process that does not involve the mental rotation of the object, but rather relies solely on changes in the observer’s perceptual perspective. Similarly, Clements (1998) defined spatial orientation as the ability to understand and use relationships among different positions in space relative to one’s own location. Contero et al. (2005) also described spatial orientation as the capacity to mentally visualize how an object would appear from another perspective. More recent studies in the literature conceptualize spatial orientation as the ability to perceive changes in the orientations of visual stimuli and to accurately interpret these changes in relation to viewpoint (Pittalis and Christou, 2010, p. 195; Risma et al., 2013, p. 75; Ung et al., 2016).

### 2.3 Cognitive processes of spatial ability

The cognitive processes discussed in this section were determined on the basis of the classification frameworks, task types, and mental operation descriptions reported in the spatial ability literature. Previous studies have classified spatial ability in different ways and have described the mental operations required in spatial tasks, such as combining and separating parts, mentally rotating objects, transforming two- and three-dimensional representations, interpreting multiple views, and using plan-based or directional information (McGee, 1979; Lohman, 1979; Pellegrino et al., 1984; Linn and Petersen, 1985; Tartre, 1990; Clements, 1998; Contero et al., 2005; Pittalis and Christou, 2010). In this study, mental integration, mental decomposition, mental rotation, viewpoint-based representation, and plan-based representation are used not as separate theoretical dimensions of spatial ability, but as item-level cognitive processes represented in the SASEPS items. Therefore, items sharing the same cognitive process code may load on different factors depending on the specific spatial operation required by each item. Accordingly, similarity in representation format should not be interpreted as equivalence in the underlying cognitive process or psychometric factor structure.

#### 2.3.1 Mental integration

Mental integration refers to the process of mentally combining separate parts or subunits to form a meaningful and coherent whole. This ability involves establishing relationships among parts, understanding the functions of these parts within the whole, and mentally reconstructing the overall structure. Mental integration is particularly relevant to spatial visualization tasks that require constructing, transforming, folding, unfolding, or mentally combining two- and three-dimensional representations (Garcia-Segarra et al., 2024; Harris and Lowrie, 2024; Linn and Petersen, 1985; Lohman, 1979; Pellegrino et al., 1984).

#### 2.3.2 Mental decomposition

Mental decomposition is the process of mentally breaking down a whole into its constituent parts and examining these parts independently. This process is closely related to spatial visualization tasks involving the analysis of parts within a structure, the interpretation of nets, two-dimensional representations of three-dimensional objects, and the reorganization of spatial representations (Clements, 1998; Fujita et al., 2020; Linn and Petersen, 1985; Patahuddin et al., 2022; Pellegrino et al., 1984; Pittalis and Christou, 2010).

#### 2.3.3 Mental rotation

Mental rotation is the ability to perceive an object or shape by mentally rotating it around different directions, angles, or axes and to compare the new views resulting from these transformations. This process involves evaluating whether the structural properties of the object are preserved throughout the transformation and establishing equivalence between representations across different orientations. Mental rotation has been widely discussed as a central mental operation in spatial ability research and is particularly important in three-dimensional spatial thinking and object transformation tasks (Lohman, 1979; Linn and Petersen, 1985; McGee, 1979; Pittalis and Christou, 2010; Shepard and Metzler, 1971).

#### 2.3.4 Viewpoint-based representation

Viewpoint-based representation refers to the ability to perceive, interpret, and mentally relate representations of an object obtained from different viewpoints (e.g., top, side, front). This ability requires understanding how a single object is represented from multiple perspectives and integrating these representations within a coherent spatial framework. It is particularly relevant to spatial orientation tasks, technical drawings, and multiple-view representations that require individuals to coordinate different perspectives of the same object (Clements, 1998; Contero et al., 2005; McGee, 1979; Pittalis and Christou, 2010; Tartre, 1990).

#### 2.3.5 Plan-based representation

Plan-based representation refers to the use of directional, positional, route-based, and plan-based information to mentally represent spatial environments and three-dimensional structures. In this process, individuals interpret two-dimensional plans, routes, illustrated instructions, or schematic spatial representations and mentally relate them to corresponding spatial configurations. Plan-based representation reflects the ability to translate symbolic, schematic, or real-life spatial information into coherent mental representations and is closely related to spatial orientation tasks involving position, direction, route use, and schematic representations of space (Clements, 1998; Freina and Ott, 2014; Lowrie and Logan, 2018; Tartre, 1990).

### 2.4 Spatial self-efficacy

One of the key variables considered to influence the development and effective use of spatial ability is an individual’s spatial self-efficacy perception. Within the framework of social learning theory, self-efficacy is defined as an individual’s belief in their capacity to plan, organize, and successfully execute the actions required to achieve a particular level of performance (Bandura, 1982). From this perspective, self-efficacy reflects individuals’ subjective evaluations of how effectively they can utilize their cognitive capacities, rather than merely the level of cognitive capacity they possess. In the present study, this task-specific self-evaluation is referred to as spatial self-efficacy perception, in line with the focus and title of the developed scale.

Self-efficacy functions as a fundamental cognitive–behavioral construct that directly influences individuals’ task choices, the effort they expend, and their persistence and resilience when confronted with difficulties (Lent et al., 1994). According to Bandura (2001), self-efficacy beliefs are derived from four primary sources: mastery experiences, vicarious experiences acquired through observing others, verbal persuasion, and individuals’ psychological and affective states. Jinks and Morgan (1996) conceptualize self-efficacy as the extent to which individuals feel competent and confident while performing a given action. Within this framework, self-efficacy is regarded as a dynamic construct grounded not only in cognitive competence itself, but also in individuals’ perceptions of their ability to effectively employ that competence.

In this context, spatial self-efficacy perception refers to individual’s perceived competence in successfully performing two- and three-dimensional spatial tasks. In other words, spatial self-efficacy reflects individuals’ level of confidence in carrying out tasks that require spatial visualization, spatial relations, and spatial orientation (Dokumacı-Sütçü, 2019). Given the multi-step, dynamic, and cognitively complex nature of spatial tasks, it can be argued that individuals’ performance in such tasks is closely related not only to their level of spatial ability, but also to their self-efficacy perceptions regarding their capacity to effectively utilize these abilities.

Therefore, identifying spatial self-efficacy plays a critical role in making sense of and interpreting individuals’ spatial performance. Particularly for preservice teachers, spatial self-efficacy may influence the selection of representations used in instruction, problem-solving approaches, and the quality of learning experiences provided to students. In this regard, examining the relationship between spatial self-efficacy and visualization performance together offers significant contributions to a holistic understanding of the cognitive and affective dimensions of spatial thinking.

### 2.5 Significance and purpose of the study

Although previous studies on spatial ability have largely focused on performance-based measures, including spatial visualization, mental rotation, and spatial orientation tasks, the present research offers an original perspective by examining spatial ability not only through performance-based measurement but also within the framework of a task-based self-efficacy perception construct (Guay, 1976; Linn and Petersen, 1985; Yoon, 2011; Dokumacı-Sütçü, 2019; Morawietz et al., 2024). The SASEPS developed in this study addresses two- and three-dimensional spatial visualization, spatial relations, and spatial orientation within an integrated structure, enabling a multidimensional assessment of preservice teachers’ spatial ability self-efficacy perceptions. Spatial visualization is considered one of the core cognitive abilities employed by individuals while performing spatial tasks, whereas visualization performance is regarded as the behavioral manifestation of this ability as measured through standardized assessment instruments. This distinction makes it possible to examine the relationship between individuals’ self-evaluations and their objective performance.

In this context, the present study complements the limited body of literature by examining the relationship between spatial self-efficacy and actual visualization performance within a single research framework. Considering the critical role of teachers—who play a central part in fostering and developing spatial skills at early ages through curricula—and the importance of possessing high levels of spatial self-efficacy (Abay et al., 2018), identifying preservice teachers’ spatial self-efficacy perception levels and examining these levels in relation to various variables are regarded as crucial for both teacher education and the effectiveness of instructional programs.

This study adopts a holistic perspective on spatial ability by integrating preservice teachers’ spatial self-efficacy perceptions with performance-based measurements, addressing spatial ability through both cognitive and affective dimensions. Accordingly, a valid and reliable instrument was developed to assess spatial self-efficacy, and the relationships between preservice teachers’ spatial self-efficacy perception levels and their visualization performance, as well as differences across selected variables, were examined. In addition to examining the relationship between spatial self-efficacy perception and visualization performance, the present study also considers gender and department as relevant variables. Gender has frequently been examined in spatial ability research, particularly in relation to spatial visualization and mental rotation tasks; however, previous findings suggest that gender-related differences are not uniform and may vary depending on task type, learning experience, and educational context (Linn and Petersen, 1985; McGee, 1979; Rafi et al., 2008; Uttal et al., 2013). Department is also considered relevant because preservice teachers enrolled in different teacher education programs may encounter different levels of spatially demanding content, such as geometry, visual representations, maps, diagrams, three-dimensional objects, and plan-based tasks. Previous studies have emphasized that spatial thinking is closely related to disciplinary experiences and the types of representations and spatial tasks encountered in learning environments (Atit et al., 2020; Lowrie and Logan, 2018; Wai et al., 2009). Therefore, examining whether preservice teachers’ spatial self-efficacy perceptions differ according to gender and department is consistent with the aim of understanding spatial self-efficacy within the context of teacher education. Accordingly, the following research questions were addressed in this study:

1 Does the scale developed to measure preservice teachers’ spatial ability self-efficacy perceptions have adequate psychometric properties in terms of validity and reliability?
2 Do preservice teachers’ spatial ability self-efficacy perception levels differ significantly according to gender and the department in which they are enrolled?
3 Is there a statistically significant relationship between preservice teachers’ spatial ability self-efficacy perception levels and their visualization performance?

## 3 Method

This study adopts a holistic perspective on spatial ability by integrating preservice teachers’ spatial self-efficacy perceptions with performance-based visualization measures. Employing a quantitative research approach, the study was designed as a scale development and application study consisting of two main phases: scale development/validation and application. In the scale development/validation phase, a valid and reliable measurement instrument was developed to assess preservice teachers’ self-efficacy perceptions regarding spatial ability. In the application phase, differences in spatial self-efficacy perceptions across selected demographic variables were examined, and the relationship between spatial self-efficacy perceptions and visualization performance was investigated. The scale development process consisted of a comprehensive literature review, item pool generation, expert evaluation, pilot testing, and the examination of validity and reliability, in line with the procedures recommended by DeVellis and Thorpe (2021). To determine preservice teachers’ spatial ability self-efficacy perceptions and visualization performance levels, a descriptive survey design was employed. The relationships between spatial self-efficacy and visualization performance were examined using a correlational research design. Furthermore, differences in preservice teachers’ spatial self-efficacy perceptions according to gender and department were investigated through a causal-comparative research design.

### 3.1 Participants

The scale development and implementation phases of the study involved different numbers of experts and participants. Participants were selected using a simple random sampling method. Prior to data collection, all participants were informed about the purpose, scope, and significance of the study, and informed consent was obtained. Participation was entirely voluntary. The distribution of experts and participants who contributed to the research process is presented in Table 2. As shown in Table 2, separate participant groups contributed to each stage of the study and to the analyses conducted for the research questions. Thus, the data for the scale development, validation, reliability, and implementation processes were obtained from independent groups.

**Table 2:** Distribution of experts and participants.

Research Questions	Process	Experts / participants
Research Question 1	Content validity	Expert Review: *3 academics and 2 mathematics teachers*
		Pilot Study Item Clarity Assessment: *34 preservice teachers*
	Construct validity	Main Administration Exploratory Factor Analysis: *378 preservice teachers* Confirmatory Factor Analysis: *140 preservice teachers*
	Reliability	Internal Consistency: *378 preservice teachers*
Research Question 2		*310 preservice teachers*
Research Question 3		*50 preservice teachers*

### 3.2 Data collection and analysis

To assess preservice teachers’ self-efficacy perceptions regarding spatial ability, the SASEPS, a 5-point Likert-type instrument developed within the scope of the present study, was employed (Appendix 1). Although Likert-type items are ordinal in nature, composite scores derived from multiple Likert-type items, such as total scale scores and factor scores, are commonly treated as continuous variables in educational and psychological research when appropriate distributional assumptions are examined (Carifio and Perla, 2008; Norman, 2010; Sullivan and Artino, 2013). In the present study, the analyses were conducted using the scale score and factor scores derived from multiple SASEPS items rather than single-item responses. Therefore, these composite scores were treated as continuous variables for statistical analysis.

The scale is grounded in a multidimensional structure comprising three factors: spatial visualization, spatial relations, and spatial orientation. The final version of the scale consists of 35 items. Internal consistency reliability analyses yielded a Cronbach’s alpha coefficient of 0.960 for the total scale, indicating excellent reliability. The alpha coefficients for the subscales were 0.956 for spatial visualization, 0.872 for spatial relations, and 0.769 for spatial orientation, reflecting acceptable to high levels of internal consistency.

To examine the relationship between preservice teachers’ self-efficacy perceptions and their visualization performance, the Revised Purdue Spatial Visualization Tests: Visualization of Rotations (Revised PSVT:R) was employed (Appendix 2). The test was originally developed by Guay (1976) and later revised by Yoon (2011). The Revised PSVT:R consists of 30 items and has demonstrated satisfactory reliability, with a reported reliability coefficient of 0.862.

Within the scope of the present study, all scale scores were rounded to two decimal places prior to interpretation. Accordingly, score interpretations for both the SASEPS and the Revised PSVT:R were categorized based on equal interval classification as follows: 1.00–1.80 (very low), 1.81–2.60 (low), 2.61–3.40 (moderate), 3.41–4.20 (high), and 4.21–5.00 (very high).

#### 3.2.1 Data collection and analysis procedures for research question 1

At the initial stage of the study, a scale was developed to assess preservice teachers’ self-efficacy perceptions regarding spatial ability. The scale development process was systematically structured in line with established psychometric principles and consisted of several sequential steps: literature review, item pool development, expert review, pilot study, main administration, and the derivation of the final scale.

During the scale development process, a comprehensive review of the literature on spatial ability was first conducted, resulting in an initial item pool consisting of 65 items. Subsequently, expert opinions were obtained from three faculty members specializing in mathematics education and two mathematics teachers. In this stage, the experts evaluated the extent to which each item represented the purpose of the study using a four-point rating scale ranging from 1 (lowest) to 4 (highest). The content validity ratio of each item was calculated using the Davis technique. Based on this analysis, seven items (Items 57, 58, 60, 62, 63, 64, and 65) with content validity ratios below 0.80 were removed from the draft scale, and the pilot testing phase was conducted with the remaining 58 items. During the pilot study, the items were administered to 34 preservice teachers to assess the clarity and comprehensibility of the item wording in terms of language and meaning. Following the pilot study, the main administration phase was conducted. The scale, formatted as a 5-point Likert-type instrument ranging from 1 (strongly disagree) to 5 (strongly agree), was administered to 406 preservice teachers. Prior to analysis, data screening procedures were performed to identify potential outliers for each item using the Outliers option and standardized *z*-scores. Twelve cases with z-scores greater than +3 or less than −3 were identified and removed from the dataset. Normality analyses were then conducted on the remaining 394 cases by examining histograms, boxplots, and normal probability plots. Subsequently, item-level outliers were examined and removed individually, and the analyses were continued with a final sample of 378 cases. The distributions were evaluated using descriptive, graphical, and statistical procedures. The measures of central tendency, including the mean, mode, and median, were found to be closely aligned. In addition, the skewness and kurtosis coefficients for each item did not deviate substantially from the acceptable range of −1 to +1 (Field, 2009; Morgan et al., 2004). Although the Kolmogorov–Smirnov test produced statistically significant results (*p* < 0.05), this result was interpreted with caution because normality decisions should not rely on a single test alone. Instead, Kolmogorov–Smirnov test results should be evaluated together with descriptive and graphical methods when examining the normality of distributions, particularly in relatively large samples (Abbott, 2011; McKillup, 2012; Stevens, 2009). Therefore, the Kolmogorov–Smirnov results were considered together with the mean, mode, median, skewness and kurtosis coefficients, histograms, boxplots, and Q–Q plots. Based on this combined evaluation, the data were considered to approximate a normal distribution.

Following these preliminary analyses, reliability analysis and both exploratory factor analysis (EFA) and confirmatory factor analysis (CFA) were conducted for the 58-item draft scale. The EFA was performed using SPSS version 25 with data collected from 406 preservice teachers, whereas the CFA was conducted using the LISREL 8.80 software package with data obtained from 150 preservice teachers. After data screening and normality assessments, the EFA was carried out with a final sample of 378 cases, and the CFA was conducted with 140 cases. Additional evidence for convergent and discriminant validity was examined using composite reliability (CR), average variance extracted (AVE), the square root of AVE, inter-factor correlations, and the heterotrait–monotrait ratio of correlations (HTMT). CR was used to evaluate the internal consistency of the latent factors, whereas AVE was used to assess the extent to which the indicators of each factor shared variance explained by the corresponding latent construct. In the literature, CR values above 0.70 are generally considered satisfactory, and AVE values of 0.50 or higher are commonly recommended as evidence of convergent validity (Fornell and Larcker, 1981; Hair et al., 2017). However, Fornell and Larcker (1981) indicate that when AVE is below 0.50 but composite reliability is adequate, the convergent validity of a construct may still be considered acceptable. Similarly, Pervan et al. (2018) reported that AVE values below 0.50 can be interpreted as acceptable when accompanied by CR values above 0.60. Therefore, AVE values should be evaluated together with CR values, standardized factor loadings, and the theoretical relevance of the retained items. Discriminant validity was evaluated using the Fornell–Larcker criterion, inter-factor correlations, and HTMT ratios. According to the Fornell–Larcker criterion, the square root of AVE for each construct should be greater than its correlations with other constructs. HTMT, on the other hand, provides a more sensitive assessment of whether conceptually related constructs are empirically distinguishable (Fornell and Larcker, 1981; Henseler et al., 2015). Detailed results of the reliability analysis as well as the EFA and CFA procedures are presented in the Findings.

#### 3.2.2 Data collection and analysis procedures for research question 2

Following the completion of the development process of SASEPS, the final version of the scale was administered to 336 preservice teachers, and the collected data were entered into SPSS version 25. After data screening and normality analyses, a final dataset consisting of 310 cases was obtained, and subsequent analyses indicated that the data were normally distributed. The results of the descriptive statistics are presented in Table 3.

**Table 3:** Descriptive statistics for the distribution of the data.

Factor/Scale	Mode	Median	$\bar{X}$	SD	Skewness	Kurtosis	Min	Max	Kolmogorov Smirnov
SV	3.45	3.46	3.55	0.61	0.03	0.46	2.00	5.00	0.00
SR	3.00	3.17	3.28	0.73	0.60	−0.04	1.83	5.00	0.00
SO	3.20	3.80	3.74	0.62	−0.14	0.18	2.00	5.00	0.00
SASEPS	3.40	3.40	3.53	0.56	0.56	0.14	2.42	4.97	0.00

$\bar{X}$ : Mean; SD: Standard Deviation; SV: Spatial Visualization; SR: Spatial Relations; SO: Spatial Orientation; SASEPS: Spatial Ability Self-Efficacy Perception Scale.

The study examined preservice teachers’ self-efficacy perceptions of spatial ability and whether these perceptions differed by gender and department of study. Gender differences were analyzed using an independent samples t-test at the 0.05 significance level, with the equality of variances assessed via Levene’s test and considered in the interpretation of results (Kuzu, 2022). Differences by department of study were examined using a one-way ANOVA at the 0.05 significance level. For statistically significant results, *post hoc* analyses were conducted, and since the homogeneity of variances assumption was violated (*p* < 0.05), the Games–Howell multiple comparison procedure was applied (Kuzu, 2022). Regarding the second sub-problem, based on data from 310 preservice teachers, Cronbach’s alpha was 0.946 for the overall scale and 0.943, 0.842, and 0.650 for its factors, indicating acceptable to high reliability (Awang, 2012; DeVellis and Thorpe, 2021).

#### 3.2.3 Data collection and analysis procedures for research question 3

In this part of the study, the relationship between preservice teachers’ self-efficacy perceptions of spatial ability and their visualization performance was examined with the participation of 50 preservice teachers. SASEPS developed within the scope of this study and the Revised PSVT:R, originally developed by Guay (1976) and later revised by Yoon (2011), were administered to the participants during the same data collection phase. The Revised PSVT:R, which consists of 30 five-option multiple-choice items requiring the mental rotation of three-dimensional objects, was administered under standard classroom conditions. Participants completed the test individually and did not receive any instructional support or feedback during the administration. First, the reliability of the Revised PSVT:R was examined. For scoring the Revised PSVT:R, correct responses were coded as 5, whereas incorrect or blank responses were coded as 1 to align the performance score metric with the 1–5 response range of the SASEPS. This coding represents a linear transformation of conventional dichotomous scoring and therefore does not alter the rank order of participants or the magnitude of Pearson correlations. Accordingly, the Revised PSVT:R mean score should be interpreted as a transformed performance score on a 1–5 metric rather than as a proportion-correct score. In this process, the KR-20 reliability coefficient was calculated as 0.834 using the Test Analysis Program (TAP). Although the test demonstrated a high reliability coefficient, item analysis revealed that five items (Items 1, 2, 3, 4, and 30) had item discrimination indices of 0.19, 0.19, 0.18, 0.12, and 0.08, respectively. Starting from the lowest index, these items were sequentially removed from the test. Since no improvement was observed and the discrimination indices continued to remain below 0.20, these five items were excluded from further analyses within the scope of this study. For the remaining 25 items, item difficulty indices ranged between 0.32 and 0.68, with an average difficulty index of 0.527. Item discrimination indices ranged from 0.29 to 0.74, with an average discrimination index of 0.510. These results indicate that the test has a moderate level of difficulty and very good item discrimination. In addition, point-biserial correlation coefficients were examined to assess the internal validity of the items. The average point-biserial correlation coefficient was found to be 0.441, indicating an acceptable level of internal validity. The KR-20 reliability coefficient for the modified, study-specific 25-item version of the Revised PSVT:R was calculated as 0.828. Finally, preservice teachers’ visualization performance scores were also found to be normally distributed, and the descriptive statistics were within acceptable ranges (Table 4).

**Table 4:** Descriptive statistics for the distribution of the data.

Test	Mode	Median	$\bar{X}$	SD	Skewness	Kurtosis	Min	Max	Kolmogorov Smirnov
Revised PSVT:R	2.76	2.92	3.10	0.87	0.210	−1.06	1.64	4.68	0.02

$\bar{X}$ : Mean; SD: Standard Deviation.

As a result, the data obtained from the SASEPS and the Revised PSVT:R were entered into SPSS 25, and the relationship between preservice teachers’ self-efficacy perceptions of spatial ability and their visualization performance was examined. Because visualization performance data were available for a smaller subgroup of 50 preservice teachers, the analysis examining the relationship between spatial self-efficacy perceptions and visualization performance was treated as exploratory. Accordingly, *r*^2^ values were calculated to indicate shared variance, and a *post hoc* power analysis was conducted to evaluate the statistical sensitivity of the exploratory correlation analyses. Pearson correlation analysis was then conducted to examine the association between spatial self-efficacy perceptions and visualization performance. Before conducting Pearson correlation analysis, the relevant assumptions were examined. The SASEPS total and subscale scores and visualization performance scores were treated as continuous variables. The normality of the distributions was evaluated using descriptive, graphical, and statistical procedures, and the data were considered to a normal distribution based on the combined evaluation of these indicators. In addition, scatterplots were examined to assess the linearity of the relationships between variables and to check for influential outliers. The visual inspection indicated that the relationships were linear and that there were no extreme outliers that would substantially distort the correlation coefficients. Therefore, Pearson correlation analysis was considered appropriate for examining the relationships between spatial self-efficacy perceptions and visualization performance.

In addition to statistical significance, effect sizes were calculated to evaluate the practical significance of the findings. Since statistically significant differences do not necessarily indicate practically meaningful differences, effect size statistics were used to support the interpretation of the results. Cohen’s *d* values are interpreted as follows: 0.00 ≤ *d* < 0.20: very small, 0.20 ≤ *d* < 0.50: small, 0.50 ≤ *d* < 0.80: medium, 0.80 ≤ *d* < 1.20: large, 1.20 ≤ *d*: very large effect size. Similarly, the η^2^ value is interpreted as 0.01 ≤ η^2^ < 0.06: small, 0.06 ≤ η^2^ < 0.14: medium, and 0.14 ≤ η^2^: large effect size (Cohen, 1988).

## 4 Findings

### 4.1 Findings for research question 1

In the scale development process, a comprehensive literature review was first conducted, an item pool was generated, expert opinions were obtained, and a pilot study was carried out. Subsequently, data screening and normality analyses were performed, after which reliability analysis and the exploratory factor analysis (EFA) and confirmatory factor analysis (CFA) procedures were conducted for the 58-item draft scale.

Initially, since it is recommended that item–total correlation coefficients exceed 0.30 (DeVellis and Thorpe, 2021), the item–total correlations of all items in the 58-item draft scale were examined, and it was found that none of the items had a correlation value below 0.30. Among the retained items, Item 32 had the lowest item–total correlation (ITC = 0.369); however, this value remained above the recommended threshold. Therefore, the item was not interpreted as tapping a distinct construct and was retained because it also showed an adequate factor loading (FL = 0.705) and represented a theoretically relevant plan-based spatial orientation task in an everyday spatial context. Following this step, EFA was conducted as the first procedure to examine the construct validity of the scale. The suitability of the data set for EFA and the adequacy of the sample size were evaluated using the Kaiser–Meyer–Olkin (KMO) measure and Bartlett’s test of sphericity. In the present study, the KMO value was found to be 0.924, and the Bartlett’s test of sphericity yielded a significant result (*p* < 0.01), indicating that the data were suitable for factor analysis (Kaiser, 1970).

Since the scale was assumed to have a multifactor structure, factor analysis was conducted using the Varimax orthogonal rotation method. Items with factor loadings below 0.50 were considered inappropriate and were therefore removed from the scale; accordingly, four items (45, 46, 51, and 53) were excluded. In addition, it has been emphasized that cross-loading items with differences of less than 0.10 between factor loadings should also be removed (Caliskan et al., 2017), and thus two items (14 and 16) were excluded from the scale. Furthermore, Field (2009) recommends that communalities, which represent the proportion of variance in each variable explained by the factors, should be greater than 0.20. In the present analysis, all communality values were found to exceed 0.20, confirming that each item shared a sufficient amount of common variance with the other items.

Moreover, 13 items (5, 10, 15, 19, 28, 30, 31, 32, 33, 34, 35, 38, and 52) were removed because they displayed unstable or theoretically uninterpretable loading patterns across the emerging factors and did not contribute to a conceptually coherent factor solution. In addition, four items (11, 12, 39, and 56) did not statistically disrupt the emerging factor solution; however, their item content and loading patterns were not sufficiently consistent with the theoretically expected domains based on expert judgment. Therefore, these items were also excluded from the final form. For the remaining 35-item draft scale, eigenvalues, percentage of variance explained, and the scree plot were used to determine the number of factors that could best represent the relationships among the items. In this study, the first factor had an eigenvalue of 15.763 and explained 45.038% of the total variance. The second factor had an eigenvalue of 2.306 and accounted for 6.587% of the variance. The third factor had an eigenvalue of 1.778, explaining 5.081% of the variance. The fourth factor yielded an eigenvalue of 1.607 and explained 4.590% of the variance, whereas the fifth and final factor had an eigenvalue of 1.166 and explained 3.332% of the variance. The total variance explained by the scale was 64.628% in the five-factor solution and 61.296% in the four-factor solution. The analysis revealed that the items clustered primarily within the first four factors with high factor loadings. Although some items also loaded on the fifth factor, these loadings were relatively low, and the differences between the loadings on the fifth factor and those on the first four factors exceeded 0.10. Therefore, it was concluded that no items were retained in the fifth factor, and the four-factor empirical EFA solution was retained for interpretation. The total variance explained by the scale was 61.296%, which is considered adequate when it falls between 40 and 60%, and high when it exceeds 60% (Awang, 2012). The eigenvalues and explained variance ratios of the factors obtained from the EFA are presented in Table 5.

**Table 5:** Initial eigenvalues and explained variance of the EFA factors.

Component	Initial eigenvalues			Extraction sums of squared loadings		
	Total	% of variance	Cumulative %	Total	% of variance	Cumulative %
1	15.763	45.038	45.038	15.763	45.038	45.038
2	2.306	6.587	51.625	2.306	6.587	51.625
3	1.778	5.081	56.706	1.778	5.081	56.706
4	1.607	4.590	61.296	1.607	4.590	61.296
5	1.166	3.332	64.628	1.166	3.332	64.628
6	0.974	2.782	67.411			
⋮	⋮	⋮	⋮			
35	0.072	0.207	100.000			

Although five factors had eigenvalues greater than 1, the fifth factor was not retained because no items were ultimately assigned to this factor. Therefore, the four-factor empirical EFA solution was retained for interpretation and explained 61.296% of the total variance. This solution was subsequently interpreted within a three-factor theoretical structure, with two-dimensional and three-dimensional spatial visualization retained as sub-factors of spatial visualization.

After the fifth factor was excluded, the four-factor empirical EFA solution was interpreted in terms of item content and theoretical coherence. This four-factor solution was also supported by the scree plot of eigenvalues. An examination of the items loading on the first factor led to labeling this factor as “two-dimensional spatial visualization,” whereas the items loading on the second factor were grouped under “three-dimensional spatial visualization.” Analysis of the items in the third factor indicated that this factor corresponded to “spatial relations,” and the items loading on the fourth factor were labeled as “spatial orientation.” However, the first and second factors were not interpreted as independent theoretical dimensions of spatial ability. Rather, consistent with expert opinions and the theoretical framework adopted in this study, these factors were considered closely related subdomains of the broader spatial visualization construct. In factor analysis, the interpretation and naming of factors should be informed not only by statistical criteria but also by the theoretical meaning and content coherence of the items (Tabachnick and Fidell, 2007). Accordingly, “two-dimensional spatial visualization” and “three-dimensional spatial visualization” were retained as sub-factors under the spatial visualization dimension. Thus, although the analysis initially supported a four-factor empirical EFA solution, the final scale was reported within a three-factor theoretical structure consisting of spatial visualization, spatial relations, and spatial orientation. This distinction was also clarified in Appendix 1, where two-dimensional spatial visualization and three-dimensional spatial visualization were presented as sub-factors under spatial visualization. Cronbach’s alpha reliability coefficients calculated to assess the reliability of the scale, based on data obtained from 378 participants, were found to be 0.960 for the overall scale and 0.956, 0.872, and 0.769 for the respective factors. These values indicate that the scale demonstrates a high level of reliability both at the overall level and across individual factors (Awang, 2012; DeVellis and Thorpe, 2021). The results of the factor analysis, including the rotated factor matrix, Cronbach’s alpha reliability coefficients, and the items removed during the analysis process, are presented in detail in Appendix 1.

Following the exploratory factor analysis, the scale—reported within a three-factor theoretical structure—was administered to 150 preservice teachers, and confirmatory factor analysis (CFA) was conducted. After data screening and normality assessments, the CFA was performed using data from 140 participants. According to Kline (2023), sample sizes around 100–200 participants can be considered acceptable for confirmatory factor analysis when factor loadings are adequate and the model structure is well specified. The results of the CFA conducted with 35 items indicated that all t-values were statistically significant at the 0.05 level, factor loadings were not below 0.30, and the error variance values of the standardized solutions did not exceed 0.90. The evaluation of model fit indices was based on commonly accepted cutoff criteria for CFI, S-RMR, χ^2^/df, and RMSEA as recommended in the literature (Hu and Bentler, 1999; Kline, 2023). Within this framework, the CFI, S-RMR, and χ^2^/df values were found to be within acceptable ranges, whereas the RMSEA value initially exceeded the acceptable threshold. Therefore, modification indices were examined, and theoretically justifiable correlations between certain error terms within the same factor were allowed. In the overall 35-item CFA model, 23 within-factor error covariances were specified based on modification indices. These covariances were allowed only between items belonging to the same factor and were justified by similarities in item wording, task format, or the cognitive process represented by the items. After these modifications, the overall model fit improved from χ^2^/df = 3.749, CFI = 0.92, S-RMR = 0.092, and RMSEA = 0.141, 90% CI [0.13, 0.15] to χ^2^/df = 2.298, CFI = 0.95, S-RMR = 0.083, and RMSEA = 0.097, 90% CI [0.090, 0.100]. These modifications improved the overall model fit without altering the theoretical structure of the scale, and all fit indices subsequently reached acceptable levels.

In addition, a separate CFA was conducted for the 24 items loading under the spatial visualization dimension to further examine whether two-dimensional spatial visualization and three-dimensional spatial visualization could be retained as theoretically meaningful sub-factors of this broader dimension. This analysis indicated statistically significant t-values, factor loadings above 0.30, and error variance values below 0.90. In the 24-item CFA model, 12 within-factor error covariances were specified based on modification indices. These covariances were allowed only between items belonging to the same factor and were justified by similarities in item wording, task format, or the cognitive process represented by the items. After these modifications, the model fit improved from χ^2^/df = 3.129, CFI = 0.94, S-RMR = 0.071, and RMSEA = 0.124, 90% CI [0.11, 0.13] to χ^2^/df = 2.136, CFI = 0.97, S-RMR = 0.064, and RMSEA = 0.090, 90% CI [0.080, 0.100]. After applying the modification procedure, the fit indices for the spatial visualization factor were also found to be within acceptable ranges. The post-modification CFA results for both the overall scale and the spatial visualization factor are presented in Table 6.

**Table 6:** CFA results for SASEPS and spatial visualization factor.

Fit indices	Excellent fit range	Acceptable fit range	SASEPS		SVF		PoM results for SASEPS and SV
			PM	PoM	PM	PoM	
*X*^2^/*df*	≤3.00	3.00–5.00	3.749	2.298	3.129	2.136	Excellent
RMSEA	≤0.05	0.05–0.10	0.141	0.097	0.124	0.090	Good
S-RMR	≤0.05	0.05–0.10	0.092	0.083	0.071	0.064	Good
CFI	≥0.95	0.90–0.95	0.92	0.95	0.94	0.97	Excellent

PM: Pre-Modification; PoM: Post-Modification; SVF: Spatial Visualization Factor.

In the present study, the CR values were 0.957 for spatial visualization, 0.818 for spatial relations, and 0.822 for spatial orientation, indicating satisfactory composite reliability for all three factors. The AVE values were 0.492, 0.476, and 0.440, respectively. Although these values were slightly below the conventional 0.50 threshold, they were close to the recommended criterion for spatial visualization and spatial relations and were accompanied by satisfactory CR values across all factors. Thus, the convergent validity evidence was considered acceptable when evaluated together with the CR values, statistically significant factor loadings, and the theoretical contribution of the items. In this study, the square roots of AVE were 0.701 for spatial visualization, 0.690 for spatial relations, and 0.663 for spatial orientation. Inter-factor correlations ranged from 0.512 to 0.694, indicating that the factors were positively related but not redundant. More importantly, the HTMT values ranged from 0.629 to 0.799, remaining below the conservative 0.85 threshold recommended for establishing discriminant validity. Taken together, these findings indicate that the three factors of the SASEPS represent theoretically related but empirically distinguishable components of spatial ability self-efficacy. Therefore, the CR, AVE, inter-factor correlation, and HTMT evidence support the convergent and discriminant validity of the three-factor structure. The CR, AVE, square root of AVE, inter-factor correlation, and HTMT results are presented in Table 7.

**Table 7:** Additional evidence for convergent and discriminant validity of the SASEPS.

Factor/Relationship	CR	AVE	Square root of AVE	Inter-factor correlation	HTMT
SV	0.957	0.492	0.701		
SR	0.818	0.476	0.690		
SO	0.822	0.440	0.663		
SV-SR				0.694	0.764
SV-SO				0.688	0.799
SR-SO				0.512	0.629

CR = Composite Reliability; AVE = Average Variance Extracted; HTMT = Heterotrait–Monotrait Ratio of Correlations; SV = Spatial Visualization; SR = Spatial Relations; SO = Spatial Orientation.

Based on the CFA results, the construct validity of the three-factor theoretical structure of the 35-item scale—comprising spatial visualization, spatial relations, and spatial orientation—was supported. In addition, the validity of the two-subdimension structure of the spatial visualization factor, consisting of two-dimensional spatial visualization and three-dimensional spatial visualization, was also supported. In light of these findings, a valid and reliable measurement instrument titled the Spatial Ability Self-Efficacy Perception Scale (SASEPS) was developed. The scale consists of 35 items rated on a 5-point Likert-type scale and demonstrated a high level of overall reliability, with a Cronbach’s alpha coefficient of 0.960 (Appendix 1).

### 4.2 Findings for research question 2

This section examines whether preservice teachers’ self-efficacy perceptions regarding spatial ability differ significantly according to gender and the type of academic program in which they are enrolled. Descriptive statistics indicated that preservice teachers’ overall self-efficacy perceptions of spatial ability were at a high level ( ${\bar{X}}_{\text{SASEPS}}=3.$ 53). With respect to the subdimensions, self-efficacy perceptions related to spatial visualization ( ${\bar{X}}_{\mathrm{SV}}=3.$ 55) and spatial orientation ( ${\bar{X}}_{\mathrm{SO}}$ =3.74) were found to be high, whereas perceptions related to spatial relations were at a moderate level ( ${\bar{X}}_{\mathrm{SR}}=3.28$ ). Detailed descriptive statistics are presented in Table 3. An independent-samples t-test was conducted to examine whether preservice teachers’ self-efficacy perceptions of spatial ability differed significantly by gender, and the results are presented in Table 8.

**Table 8:** Independent samples *t*-test results for spatial ability self-efficacy by gender.

Factor/Scale	Gender	*N*	$\bar{X}$	SD	*t*	*p*	*d*
SV	Female	265	3.56	0.61	0.627	0.531	0.12
	Male	45	3.49	0.54			
SR	Female	265	3.28	0.74	−0.087	0.931	0.01
	Male	45	3.29	0.61			
SO	Female	265	3.75	0.62	0.618	0.537	0.11
	Male	45	3.68	0.63			
SASEPS	Female	265	3.53	0.56	0.548	0.584	0.08
	Male	45	3.49	0.49			

^*^ p < 0.05; $\bar{X}$ : Mean; SD: Standard Deviation.

The findings indicated that there was no statistically significant difference in self-efficacy perceptions of spatial ability based on gender (*p* > 0.05). Nevertheless, although the difference was not statistically significant, female preservice teachers demonstrated higher levels of spatial ability self-efficacy than their male counterparts. In addition, both gender groups exhibited high levels of spatial ability self-efficacy ( ${\bar{X}}_{\text{female}}=3.53$ ; ${\bar{X}}_{\text{male}}=3.49$ ). Effect sizes were also calculated using Cohen’s *d* to evaluate the practical significance of gender-based differences. The effect sizes were very small for spatial visualization, spatial relations, spatial orientation, and the overall SASEPS score (*d* = 0.12, 0.06, 0.11, and 0.08, respectively). These findings indicate that gender-based differences in preservice teachers’ spatial ability self-efficacy perceptions were negligible.

To examine whether preservice teachers’ self-efficacy perceptions of spatial ability differed according to their academic programs, a one-way analysis of variance (ANOVA) was conducted, and the results are presented in Table 9.

**Table 9:** ANOVA results for spatial ability self-efficacy by department.

Factor/Scale	Department	*N*	$\bar{X}$	*F*	*p*	Difference	η^2^
SV	1. Mathematics Education	91	3.79	6.20	0.000*	1 > 2 1 > 4 1 > 5	0.07
	2. Primary Education	66	3.45				
	3. Social Studies Education	49	3.53				
	4. Religious Culture and Moral Education	56	3.35				
	5. Guidance and Psychological Counseling	48	3.45				
SR	1. Mathematics Education	91	3.48	8.73	0.000*	1 > 4	0.10
	2. Primary Education	66	3.37			1 > 5	
	3. Social Studies Education	49	3.41			2 > 5	
	4. Religious Culture and Moral Education	56	3.11			3 > 4	
	5. Guidance and Psychological Counseling	48	2.83			3 > 5	
SO	1. Mathematics Education	91	4.00	9.43	0.000*	1 > 2	0.11
	2. Primary Education	66	3.67			1 > 4	
	3. Social Studies Education	49	3.85			1 > 5	
	4. Religious Culture and Moral Education	56	3.53			3 > 4	
	5. Guidance and Psychological Counseling	48	3.46			3 > 5	
SASEPS	1. Mathematics Education	91	3.77	7.98	0.000*	1 > 4 1 > 5 3 > 5	0.09
	2. Primary Education	66	3.47				
	3. Social Studies Education	49	3.56				
	4. Religious Culture and Moral Education	56	3.34				
	5. Guidance and Psychological Counseling	48	3.35				

^*^p < 0.05; $\bar{X}$ : Mean; SD: Standard Deviation.

The findings revealed statistically significant differences across academic programs with respect to the overall scale and all subdimensions (*p* < 0.05). *Post hoc* comparisons indicated that preservice teachers enrolled in Mathematics Education and Social Studies Education demonstrated significantly higher levels of spatial visualization self-efficacy than those enrolled in Primary Education, Religious Culture and Moral Education, and Guidance and Psychological Counseling programs. In the spatial relations dimension, preservice teachers in Mathematics Education exhibited significantly higher self-efficacy perceptions compared to those in Religious Culture and Moral Education and Guidance and Psychological Counseling programs. Additionally, a significant difference was found in favor of preservice teachers in the Primary Education program compared to those in Guidance and Psychological Counseling. Regarding spatial orientation, preservice teachers enrolled in Mathematics Education and Social Studies Education reported significantly higher self-efficacy perceptions than those enrolled in Primary Education, Religious Culture and Moral Education, and Guidance and Psychological Counseling programs.

In terms of the overall scale, preservice teachers in Mathematics Education and Social Studies Education demonstrated significantly higher spatial ability self-efficacy perceptions, particularly when compared with those in Religious Culture and Moral Education and Guidance and Psychological Counseling. As shown in Table 9, preservice teachers enrolled in Mathematics Education ( ${\bar{X}}_{\mathrm{ME}}=3.77$ ), Primary Education ( ${\bar{X}}_{\mathrm{PE}}=3.47$ ), and Social Studies Education ( ${\bar{X}}_{\mathrm{SSE}}=3.56$ ) reported high levels of spatial ability self-efficacy, whereas those enrolled in Religious Culture and Moral Education ( ${\bar{X}}_{\text{RCEE}}=3.34$ ) and Guidance and Psychological Counseling ( ${\bar{X}}_{\mathrm{GPC}}=3.35$ ) exhibited moderate levels of self-efficacy. Effect sizes were calculated using eta squared (η^2^) to evaluate the practical significance of department-based differences. The eta squared values indicated moderate effects for spatial visualization, spatial relations, spatial orientation, and the overall SASEPS score (η^2^ = 0.075, 0.103, 0.110, and 0.095, respectively). These results suggest that department has a meaningful practical effect on preservice teachers’ spatial ability self-efficacy perceptions, particularly for spatial orientation and spatial relations.

### 4.3 Findings for research question 3

This section examines the relationship between preservice teachers’ self-efficacy perceptions regarding spatial ability and their visualization performance. Descriptive statistics indicated that preservice teachers’ visualization performance was at a moderate level ( ${\bar{X}}_{\text{RevisedPSVT}:R}=3.10$ ). Detailed descriptive statistics are presented in Table 4. In addition, the relationship between preservice teachers’ self-efficacy perceptions and their visualization performance was examined using Pearson’s correlation analysis, and the results are presented in Table 10. An examination of Table 10 revealed a statistically significant, positive, and moderate relationship between preservice teachers’ spatial ability self-efficacy perceptions and all factors—except for the spatial orientation factor—as well as the overall scale (0.40 < *r* < 0.60; *p* < 0.05). With respect to spatial orientation, the relationship was also positive but at a weak level (0.20 < *r* < 0.40; *p* < 0.05).

**Table 10:** Relationship between spatial ability self-efficacy and visualization performance.

Test	Spatial ability self-efficacy perception											
	SV			SR			SO			SASEPS		
	*r*	*r^2^*	*p*	*r*	*r^2^*	*p*	*r*	*r^2^*	*p*	*r*	*r^2^*	*p*
Revised PSVT:R	0.581	0.338	0.000*	0.484	0.234	0.000*	0.317	0.100	0.025*	0.586	0.343	0.000*

^*^p < 0.05.

In addition to the correlation coefficients, squared correlation coefficients (r^2^) were calculated to indicate the proportion of shared variance between SASEPS scores and visualization performance. The *r*^2^ values were 0.338 for spatial visualization, 0.234 for spatial relations, 0.100 for spatial orientation, and 0.343 for the total SASEPS score. A *post hoc* power analysis was also conducted for the correlations reported under Research Question 3 using *n* = 50, *α* = 0.05, and a two-tailed test. The achieved power values were 0.998 for spatial visualization, 0.964 for spatial relations, 0.621 for spatial orientation, and 0.998 for the total SASEPS score. Although the correlations involving the total SASEPS score, spatial visualization, and spatial relations showed adequate statistical power, the power for the spatial orientation correlation was below the conventional 0.80 threshold. Therefore, the association between spatial orientation and visualization performance should be interpreted cautiously as a preliminary exploratory finding requiring replication with larger samples.

## 5 Conclusion and discussion

In this study, the Spatial Ability Self-Efficacy Perception Scale was developed to assess preservice teachers’ self-efficacy perceptions regarding spatial ability, and psychometric evidence was obtained regarding its validity and reliability. In addition, the study examined whether spatial self-efficacy perceptions differ across various variables and explored the relationship between these perceptions and visualization performance. Findings related to the first research question indicate that SASEPS was developed with a three-factor structure comprising spatial visualization, spatial relations, and spatial orientation, consistent with the multidimensional theoretical framework of spatial ability widely accepted in the literature (Lohman, 1979; Linn and Petersen, 1985; Tartre, 1990). The spatial visualization factor includes two-dimensional and three-dimensional subdimensions, reflecting the multi-stage and cognitively complex nature of spatial visualization. The spatial relations factor encompasses self-efficacy perceptions related to positional relationships between objects and mental transformations, whereas the spatial orientation factor addresses self-efficacy perceptions associated with spatial restructuring based on an individual’s perceptual perspective. The high reliability coefficients of the scale indicate that spatial self-efficacy can be measured consistently and reliably within a preservice teacher sample. However, the very high Cronbach’s alpha coefficient of the overall scale should be interpreted with caution, as very high alpha values may also indicate potential item overlap or redundancy (Tavakol and Dennick, 2011). In this study, the items were retained by considering not only internal consistency but also content validity ratios, item clarity, item-total correlations, factor loadings, cross-loadings, communality values, theoretical relevance, expert evaluations, and the representation of different spatial task types. In this respect, SASEPS contributes to the literature as a significant measurement tool that conceptualizes spatial self-efficacy within a task-based and multidimensional framework.

The findings related to the second research question revealed that preservice teachers’ spatial self-efficacy perceptions were generally high, with higher levels in the spatial visualization and spatial orientation dimensions and a moderate level in the spatial relations dimension. This pattern indicates that the dimensions of spatial ability are experienced at different levels by preservice teachers. The literature emphasizes that spatial visualization and spatial orientation are more closely associated with tasks frequently encountered in daily life and instructional contexts, whereas spatial relations involve more complex cognitive processes such as mental rotation and transformation (Lohman, 1979; Linn and Petersen, 1985; Tartre, 1990). The present findings support this theoretical distinction. Results regarding the gender variable indicated no statistically significant differences in preservice teachers’ spatial self-efficacy perceptions between female and male participants. This finding is consistent with international literature suggesting that gender differences in the spatial domain may not be decisive in terms of self-efficacy perceptions (Linn and Petersen, 1985). It can be argued that preservice teachers’ exposure to similar academic and instructional experiences contributes to the attenuation of gender-based differences. In contrast, preservice teachers’ spatial self-efficacy perceptions differed significantly according to their academic programs. Higher self-efficacy levels observed among students enrolled in Mathematics Education and Social Studies Education programs may be attributed to the more intensive use of visual representations, the concretization of abstract structures, and multiple representations in instructional processes within these programs. Conversely, in Primary Education, Religious Culture and Moral Education, and Guidance and Psychological Counseling programs, spatial thinking is often addressed implicitly and in a less systematic manner, which may have contributed to comparatively lower levels of spatial self-efficacy among preservice teachers (Clements, 1998). Overall, these findings indicate that spatial self-efficacy is a curriculum-sensitive and malleable construct. The effect size results further support this interpretation. The very small Cohen’s *d* values obtained for gender-based comparisons indicate that the differences between female and male preservice teachers were negligible not only statistically but also practically. In contrast, the moderate eta squared values obtained for department-based comparisons suggest that academic program has a meaningful practical effect on spatial ability self-efficacy perceptions. This finding supports the view that spatial self-efficacy may be shaped more by disciplinary learning experiences and exposure to spatially demanding tasks than by gender alone. This interpretation is also consistent with studies suggesting that gender differences in spatial ability are not fixed or uniform, but may vary depending on task characteristics, prior spatial experiences, and training opportunities (Linn and Petersen, 1985; Rafi et al., 2008; Uttal et al., 2013). In the present study, the absence of a meaningful gender effect may indicate that gender alone is not a decisive factor in preservice teachers’ spatial self-efficacy perceptions. Rather, spatial self-efficacy may be more closely associated with individuals’ prior experiences with spatial tasks, the frequency of exposure to visual and spatial representations, and the nature of learning opportunities provided in their academic programs. In this regard, the department-based differences are in line with research emphasizing that spatial thinking is shaped by disciplinary experiences and by the frequency with which learners encounter spatial representations, visual models, maps, diagrams, and geometry-related tasks in their academic programs (Atit et al., 2020; Lowrie and Logan, 2018; Wai et al., 2009).

Findings related to the third research question revealed positive and moderate statistically significant relationships between preservice teachers’ spatial self-efficacy perceptions and their visualization performance, both for the overall scale and for the spatial visualization and spatial relations dimensions. This result indicates that individuals’ self-efficacy perceptions regarding their ability to successfully perform spatial tasks are meaningfully associated with their actual performance. This finding is consistent with social cognitive theory, which emphasizes that self-efficacy influences individuals’ effort, persistence, strategy use, and resilience during task engagement, but does not function as the sole determinant of performance (Bandura, 1982, 2001; Lent et al., 1994). Similarly, studies focusing on spatial ability and spatial learning suggest that individuals’ confidence in performing spatial tasks may be associated with their engagement, navigation or visualization performance, and performance-related outcomes in spatial task contexts (Dokumacı-Sütçü, 2019; Miola et al., 2021; Rérat and Berger, 2026). The moderate strength of the relationships found in the present study suggests that spatial self-efficacy and visualization performance are related but not identical constructs. In other words, preservice teachers’ confidence in performing spatial tasks appears to be meaningfully associated with their actual visualization performance, yet performance is also likely influenced by other cognitive, experiential, and instructional factors. By contrast, the relatively weak relationship between self-efficacy in the spatial orientation dimension and visualization performance may be explained by the fact that this dimension involves cognitive processes based on perceptual perspective transformations rather than direct object manipulation (McGee, 1979; Tartre, 1990; Clements, 1998). Accordingly, it is expected that self-efficacy perceptions related to spatial orientation would demonstrate more limited associations with instruments that primarily assess object-based visualization performance. Overall, the findings underscore the necessity of jointly addressing the cognitive (performance) and affective (self-efficacy) dimensions of spatial ability. Preservice teachers’ strong self-efficacy perceptions regarding the execution of spatial tasks may support their current performance and may also contribute to their potential to integrate spatially demanding instructional practices into classroom environments. In this respect, the present study supports the existing literature advocating for a holistic and structured approach to spatial thinking within the context of teacher education.

## 6 Implications

This study demonstrates that, within the context of teacher education, spatial ability should be addressed not only through performance-based assessments but also in conjunction with preservice teachers’ self-efficacy perceptions regarding their ability to perform spatial tasks. This finding highlights the importance of adopting a holistic approach in teacher education programs that integrates both the cognitive and affective dimensions of spatial thinking.

Learning experiences encompassing spatial visualization, spatial relations, and spatial orientation should be systematically structured around explicit learning objectives in order to support the development of preservice teachers’ spatial self-efficacy. Engagement with two- and three-dimensional representations, the use of multiple representations, and the incorporation of tasks requiring spatial transformations within instructional processes contribute to the comprehensive support of spatial thinking.

Furthermore, technology-enhanced and practice-oriented learning environments appear to hold considerable potential for fostering spatial thinking. The use of dynamic geometry software, three-dimensional modeling tools, and digital manipulatives may enable a more systematic integration of spatial thinking, particularly within primary education programs.

From an assessment perspective, the findings suggest that spatial ability should be examined through multidimensional approaches that encompass not only performance indicators but also self-efficacy perceptions. In this regard, the SASEPS may be considered a functional instrument for monitoring the development of spatial thinking within teacher education programs.

In practical terms, SASEPS may be used as a diagnostic and monitoring tool in teacher education programs. For example, it may be administered at the beginning and end of courses involving geometry, visual representations, instructional materials, map use, or spatial reasoning in order to monitor changes in preservice teachers’ spatial self-efficacy perceptions. Teacher educators may use the total scale and subscale scores to identify areas in which preservice teachers feel less confident, such as spatial relations or spatial orientation. Based on these results, instructional activities may be designed to target specific needs through mental rotation tasks, multiple-view representations, plan interpretation, map-reading activities, 3D models, dynamic geometry software, digital manipulatives, and tasks involving the transformation between two- and three-dimensional representations.

## 7 Limitations

This study has several limitations. First, the study was conducted with preservice teachers from a single public university. Therefore, the generalizability of the findings to broader samples and different institutional contexts is limited. Second, SASEPS is a self-report instrument; therefore, participants’ responses may be influenced by subjective evaluations of their own spatial abilities and self-report bias. Third, the study employed a cross-sectional design. Therefore, the relationship between spatial self-efficacy perceptions and visualization performance should not be interpreted causally. Fourth, although the sample size used for CFA was within the acceptable range suggested in the literature under conditions of adequate factor loadings and a theoretically specified model, it should still be considered relatively limited for evaluating a 35-item scale. Therefore, although the CFA findings supported the proposed theoretical structure, they should be interpreted with this sample-size limitation in mind. Fifth, the relationship between spatial self-efficacy perceptions and visualization performance was examined with a relatively smaller subgroup of 50 preservice teachers. Therefore, the findings regarding this relationship should be interpreted as exploratory and with caution. In particular, the *post hoc* power analysis indicated that the weakest statistically significant association, namely the relationship between spatial orientation and visualization performance, had limited statistical power below the conventional 0.80 threshold. Accordingly, the observed associations, especially the spatial orientation–performance relationship, should be regarded as preliminary evidence. Finally, visualization performance was measured using a modified, study-specific 25-item version of the Revised PSVT:R from which five items with low item discrimination indices were removed. Although the modified version showed acceptable reliability and item-level functioning in the present sample, direct comparisons with studies using the complete 30-item Revised PSVT:R should be made with caution. In addition, because the Revised PSVT:R primarily focuses on mental rotation and visualization performance, other aspects of spatial performance may not have been fully captured.

## 8 Recommendations for practice and future research

Based on the findings of this study, the following recommendations are offered for teacher education practices and for future research:

First, it is recommended that spatial thinking be addressed within teacher education programs through explicit, targeted, and structured learning outcomes. The systematic integration of instructional activities that directly target spatial visualization, spatial relations, and spatial orientation into course content may strengthen preservice teachers’ spatial self-efficacy perceptions.

In primary education programs, it is particularly important to make instructional practices related to spatial thinking more visible and structured. Pedagogical frameworks that explicitly articulate the relationship between games, materials, and activity-based practices and spatial thinking may support preservice teachers’ conscious and intentional use of these skills.

In addition, it is recommended that the assessment of spatial ability employ multidimensional evaluation approaches that incorporate self-efficacy perceptions alongside performance-based measures. In this context, the SASEPS may be utilized as a diagnostic and monitoring instrument to track preservice teachers’ development in spatial thinking.

For future research, it is suggested that the relationship between spatial self-efficacy and performance be examined across different disciplines, diverse samples, and through longitudinal research designs. Moreover, experimental and mixed-methods studies investigating the indirect effects of spatial self-efficacy on teachers’ classroom practices and student learning outcomes are likely to make substantial contributions to the literature.

## Data Availability

The raw data supporting the conclusions of this article will be made available by the authors, without undue reservation.

## References

[ref1] Abay S. Tertemiz N. Gökbulut Y. (2018). Investigatıon in several variables the spatial skills of teacher candidates. Necatibey Fac. Educ. Electron. J. Sci. Math. Educ. 12, 45–62. doi: 10.17522/balikesirnef.437657

[ref2] Abbott M. L. (2011). Understanding Educational Statistics Using Microsoft Excel and SPSS. Hoboken, NJ, USA: John Wiley & Sons.

[ref3] Arıkan A. Çetin T. (2024). Psychometric properties of the spatial skills self-efficacy scale: a validity and reliability study. Think. Skills Creat. 52:101555. doi: 10.1016/j.tsc.2024.101555

[ref4] Atit K. Power J. R. Pigott T. Lee J. Geer E. A. Uttal D. H. . (2022). Examining the relations between spatial skills and mathematical performance: a meta-analysis. Psychon. Bull. Rev. 29, 699–720. doi: 10.3758/s13423-021-02012-w, 34799844

[ref5] Atit K. Uttal D. H. Stieff M. (2020). Situating space: using a discipline-focused lens to examine spatial thinking skills. Cognit. Res. Princ. Implic. 5:19. doi: 10.1186/s41235-020-00210-z, 32323024 PMC7176750

[ref6] Awang Z. (2012). Research Methodology and Data Analysis Second Edition. Shah Alam, Malaysia: UiTM Press.

[ref7] Bandura A. (1982). Self-efficacy mechanism in human agency. Am. Psychol. 37, 122–147. doi: 10.1037/0003-066x.37.2.122

[ref8] Bandura A. (2001). The changing face of psychology at the dawning of a globalization era. Can. Psychol. Psychol. Can. 42, 12–24. doi: 10.1037/h0086876

[ref9] Caliskan N. Kuzu O. Kuzu Y. (2017). The development of a behavior patterns rating scale for preservice teachers. J. Educ. Learn. 6, 130–142. doi: 10.5539/jel.v6n1p130

[ref10] Carifio J. Perla R. J. (2008). Resolving the 50-year debate around using and misusing Likert scales. Med. Educ. 42, 1150–1152. doi: 10.1111/j.1365-2923.2008.03172.x, 19120943

[ref11] Clements D. H. (1998) Geometric and Spatial Thinking in Young Children ERIC. Arlington, VA, USA. Available online at: https://eric.ed.gov/?id=ED436232 (Accessed June 13, 2026).

[ref12] Cohen J. (1988). Statistical Power Analysis for the Behavioral Sciences. 2nd Edn. Hillsdale, NJ, USA: Lawrence Erlbaum.

[ref13] Contero M. Naya F. Company P. Saorín J. L. Conesa J. (2005). Improving visualization skills in engineering education. IEEE Comput. Graph. Appl. 25, 24–31. doi: 10.1109/MCG.2005.107, 16209167

[ref14] DeVellis R. F. Thorpe C. T. (2021). Scale Development: Theory and Applications. Thousand Oaks, CA, USA: Sage.

[ref15] Dokumacı-Sütçü N. (2019). The relationship between preservice teachers’ spatial abilities and spatial ability self-reports. Mediterr. J. Educ. Res. 13, 296–315. doi: 10.29329/mjer.2019.210.16

[ref16] Erdenechimeg S. Danaa G. (2023). Some results and evaluation of training for the development of students’ spatial visualization. Embed. Selforganis. Syst. 10, 82–88. doi: 10.14464/ess.v10i7.630

[ref17] Field A. (2009). Discovering Statistics Using SPSS. London, UK: Sage.

[ref18] Fornell C. Larcker D. F. (1981). Evaluating structural equation models with unobservable variables and measurement error. J. Mark. Res. 18, 39–50. doi: 10.2307/3151312

[ref19] Freina L. Ott M. (2014) Discussing implementation choices for serious games supporting spatial and orientation skills ICERI2014 Proceedings. 5182–5191.

[ref20] Fujita T. Kondo Y. Kumakura H. Kunimune S. Jones K. (2020). Spatial reasoning skills about 2D representations of 3D geometrical shapes in grades 4 to 9. Math. Educ. Res. J. 32, 235–255. doi: 10.1007/s13394-020-00335-w

[ref21] Garcia-Segarra P. Santamarta V. Falomir Z. (2024). Educating on spatial skills using a paper-folding-and-punched-hole videogame: gameplay data analysis. Front. Educ. 9:1303932. doi: 10.3389/feduc.2024.1303932

[ref22] Guay R. B. (1976). Purdue Spatial Visualization Test. Thousand Oaks, CA, USA: Purdue Research Foundation.

[ref23] Hair J. F. Jr. Hult G. T. M. Ringle C. M. Sarstedt M. (2017). A primer on Partial least Squares Structural Equation Modeling (PLS-SEM). 2nd Edn. Thousand Oaks, CA, USA: SAGE Publications.

[ref24] Harris D. Lowrie T. (2024). “Insights from paper folding: spatial visualization processes and their link to mathematics,” in German Conference on Spatial Cognition, (Cham, Switzerland: Springer Nature Switzerland), 3–18.

[ref25] Henseler J. Ringle C. M. Sarstedt M. (2015). A new criterion for assessing discriminant validity in variance-based structural equation modeling. J. Acad. Mark. Sci. 43, 115–135. doi: 10.1007/s11747-014-0403-8

[ref26] Hu L. T. Bentler P. M. (1999). Cutoff criteria for fit indexes in covariance structure analysis: conventional criteria versus new alternatives. Struct. Equ. Model. Multidiscip. J. 6, 1–55. doi: 10.1080/10705519909540118

[ref27] Jinks J. Morgan V. L. (1996). Students' sense of academic efficacy and achievement in science: a useful new direction for research regarding scientific literacy. Electron. J. Sci. Educ. 1:n2.

[ref28] Kaiser H. F. (1970). A second-generation little jiffy. Psychometrika 35, 401–415. doi: 10.1007/bf02291817

[ref29] Kline R. B. (2023). Principles and Practice of Structural Equation Modeling. New York, NY, USA: Guilford publications.

[ref30] Kuzu Y. (2022). Ortalamalar arası farkın test edilmesi [testing the difference between the means]. In Göçer-Şahin S. Buluş M. (Eds.), Adım adım uygulamalı istatistik [Step by step applied statistics] (pp. 105–156). Ankara, Türkiye: Pegem Academy Publication.

[ref31] Lent R. Brown S. D. Hackett G. (1994). Toward a unifying social cognitive theory of career and academic interest, choice and performance. J. Vocat. Behav. 45, 79–122. doi: 10.1006/jvbe.1994.1027

[ref32] Linn M. C. Petersen A. C. (1985). Emergence and characterization of gender differences in spatial abilities: a meta-analysis. Child Dev. 56, 1479–1498. doi: 10.2307/1130467, 4075870

[ref33] Lohman D. F. (1979) Spatial Ability: A Review and Reanalysis of the Correlational Literature (Tech. Rep. No. 8). Stanford, CA, USA: Aptitude Research Project, School of Education, Stanford University.

[ref34] Lowrie T. Logan T. (2018). “The interaction between spatial reasoning constructs and mathematics understandings in elementary classrooms: the role of spatial reasoning in mathematical thought,” in Visualizing Mathematics, eds. Mix K. S. Battista M. T. (Cham, Switzerland: Springer International Publishing), 253–276.

[ref35] Maccoby E. E. Jacklin C. N. (1974). The Psychology of sex Differences. Stanford, CA, USA: Stanford University Press.

[ref36] McGee M. G. (1979). Human spatial abilities: psychometric studies and environmental, genetic, hormonal, and neurological influences. Psychol. Bull. 86, 889–918. doi: 10.1037/0033-2909.86.5.889, 386403

[ref37] McKillup S. (2012). Statistics Explained: An Introductory Guide for Life Scientists. 2nd Edn Cambridge University Press.

[ref38] Miola L. Muffato V. Meneghetti C. Pazzaglia F. (2021). Spatial learning in a virtual environment: the role of self-efficacy feedback and individual visuospatial factors. Brain Sci. 11:1185. doi: 10.3390/brainsci11091185, 34573205 PMC8467250

[ref39] Morawietz C. Dumalski N. Wissmann A. M. Wecking J. Muehlbauer T. (2024). Consistency of spatial ability performance in children, adolescents, and young adults. Front. Psychol. 15:1365941. doi: 10.3389/fpsyg.2024.1365941, 38487665 PMC10938598

[ref40] Morgan G. A. Leech N. L. Gloeckner G. W. Barrett K. C. (2004). SPSS for Introductory Statistics: Use and Interpretation. Mahwah, NJ, USA: Psychology Press.

[ref41] Norman G. (2010). Likert scales, levels of measurement and the “laws” of statistics. Adv. Health Sci. Educ. 15, 625–632. doi: 10.1007/s10459-010-9222-y, 20146096

[ref42] Patahuddin S. M. Ramful A. Lowrie T. Bholoa A. (2022). Subtleties in spatial visualization maneuvers: insights from numerical solutions. J. Math. Behav. 67:100988. doi: 10.1016/j.jmathb.2022.100988

[ref43] Pellegrino J. W. Alderton D. L. Shute V. J. (1984). Understanding spatial ability. Educ. Psychol. 19, 239–253. doi: 10.1080/00461528409529300

[ref44] Pervan M. Curak M. Pavic Kramaric T. (2018). The influence of industry characteristics and dynamic capabilities on firms’ profitability. Int. J. Financ. Stud. 6:4. doi: 10.3390/ijfs6010004

[ref45] Pittalis M. Christou C. (2010). Types of reasoning in 3D geometry thinking and their relation with spatial ability. Educ. Stud. Math. 75, 191–212. doi: 10.1007/s10649-010-9251-8

[ref46] Rafi A. Samsudin K. A. Said C. S. (2008). Training in spatial visualization: the effects of training method and gender. Educ. Technol. Soc. 11, 127–140.

[ref47] Rérat O. Berger J. L. (2026). The role of motivational beliefs and monitoring in learning spatial skills. Empir. Res. Vocat. Educ. Train. 18:3. doi: 10.1186/s40461-026-00204-z

[ref48] Risma D. A. van Eerde D. Abel M. Putri I. Ilma R. (2013) Developing students’ spatial ability through spatial visualisation and spatial orientation tasks. In Proceedings of the First South East Asia Design/Development Research (SEA-DR) International Conference, Sriwijaya University. Palembang, Indonesia.

[ref49] Shepard R. N. Metzler J. (1971). Mental rotation of three-dimensional objects. Science 171, 701–703. doi: 10.1126/science.171.3972.701 5540314

[ref50] Stevens J. P. (2009). Applied Multivariate Statistics for the Social Sciences. 5th Edn Taylor & Francis.

[ref51] Sullivan G. M. Artino A. R. Jr. (2013). Analyzing and interpreting data from Likert-type scales. J. Grad. Med. Educ. 5, 541–542. doi: 10.4300/JGME-5-4-18, 24454995 PMC3886444

[ref52] Tabachnick B. G. Fidell L. S. (2007). Using Multivariate Statistics. 5th Edn. Boston, MA, USA: Allyn & Bacon/Pearson Education.

[ref53] Tartre L. A. (1990). Spatial orientation skill and mathematical problem solving. J. Res. Math. Educ. 21, 216–229. doi: 10.2307/749375

[ref54] Tavakol M. Dennick R. (2011). Making sense of Cronbach’s alpha. Int. J. Med. Educ. 2, 53–55. doi: 10.5116/ijme.4dfb.8dfd, 28029643 PMC4205511

[ref55] Ung P. Ngowtrakula B. Chotpradita R. Thavornwong N. (2016). Spatial ability test for upper-elementary school student: confirmatory factor and normative data analysis. J. Assoc. Res. 21, 48–57.

[ref56] Uttal D. H. Meadow N. G. Tipton E. Hand L. L. Alden A. R. Warren C. . (2013). The malleability of spatial skills: a meta-analysis of training studies. Psychol. Bull. 139, 352–402. doi: 10.1037/a0028446, 22663761

[ref57] Verdine B. N. Golinkoff R. M. Hirsh-Pasek K. Newcombe N. S. Filipowicz A. T. Chang A. (2014). Deconstructing building blocks: preschoolers' spatial assembly performance relates to early mathematical skills. Child Dev. 85, 1062–1076. doi: 10.1111/cdev.12165, 24112041 PMC3962809

[ref58] Wai J. Lubinski D. Benbow C. P. (2009). Spatial ability for STEM domains: aligning over 50 years of cumulative psychological knowledge solidifies its importance. J. Educ. Psychol. 101, 817–835. doi: 10.1037/a0016127

[ref59] Yoon S. Y. (2011). Psychometric Properties of the Revised Purdue Spatial Visualization Tests: Visualization of Rotations (The Revised PSVT: R). West Lafayette, IN, USA: Purdue University.

