# Supplementary material for: The relationship between spatial ability self-efficacy and visualization performance: psychometric evidence
Source: Front Psychol. 2026 Jul 22;17:1829983. doi: 10.3389/fpsyg.2026.1829983 (PMC13437522; doi:10.3389/fpsyg.2026.1829983)
Supplement: Supplementary file 1 [file Data_sheet_1.pdf]

APPENDIX 1 SASEPS development and measurement information

TABLE A1 Factor structure of the final scale items.

No1	No2	Item	F	SF	CP	FL	ITC
7	1	I can mentally visualize and construct a rule-based pattern composed of two-dimensional shapes	SV	2DSV	MI	.798	.536
8	2	I can identify which two-dimensional shapes combine to form a two-dimensional shape.	SV	2DSV	MD	.723	.672
6	3	I can mentally visualize and draw the next step of a pattern composed of two-dimensional shapes.	SV	2DSV	MI	.717	.625
2	4	I can draw the two-dimensional projections of three-dimensional geometric objects on paper.	SV	2DSV	MI	.684	.568
9*	5*	I have difficulty decomposing a two-dimensional shape into parts and forming new two-dimensional shapes.	SV	2DSV	MD	.623	.532
4	6	I can distinguish identical two-dimensional shapes presented in different orientations.	SV	2DSV	VBR	.590	.630
3*	7*	I have difficulty drawing the required two-dimensional shapes.	SV	2DSV	VBR	.515	.503
1	8	I can mentally visualize shapes formed by the combination of two or more two-dimensional shapes.	SV	2DSV	MI	.504	.532
29	9	I can construct the net of a three-dimensional geometric solid when its closed form is given.	SV	3DSV	MI	.745	.705
20	10	I can recognize different nets of a three-dimensional geometric solid.	SV	3DSV	VBR	.734	.727
42	11	I can mentally visualize the shape of a three-dimensional object after it has been rotated.	SV	3DSV	MR	.724	.724
37	12	I can construct the net of a cube with different shapes on each face, including the shapes on its surfaces.	SV	3DSV	MI	.722	.735
24	13	I can mentally visualize and draw the next step of a pattern composed of three-dimensional objects.	SV	3DSV	MI	.719	.742
44	14	I can recognize a cube with different shapes on each face even when it is presented in different orientations.	SV	3DSV	VBR	.700	.737
21	15	I can draw the two-dimensional views of three-dimensional geometric objects from different perspectives.	SV	3DSV	MI	.695	.718
23	16	I can mentally visualize the cross-sectional surfaces of three-dimensional geometric objects.	SV	3DSV	MI	.687	.694
36	17	Given the net of a cube with different shapes on each face, I can reconstruct its closed form by considering the shapes on its surfaces.	SV	3DSV	MI	.681	.725
17	18	I can construct the closed form of three-dimensional geometric solids from their given nets.	SV	3DSV	MI	.677	.780
27	19	I can identify which shapes make up the surfaces of a three-dimensional geometric solid.	SV	3DSV	MD	.669	.764
22	20	I can draw the visual representation of three-dimensional geometric objects given their two-dimensional views from different directions.	SV	3DSV	MI	.669	.720
25	21	I can mentally visualize and construct a rule-based pattern composed of three-dimensional objects.	SV	3DSV	MI	.613	.734
13	22	I can mentally visualize geometric objects formed by the combination of two or more three-dimensional objects.	SV	3DSV	MI	.610	.773
18	23	I can distinguish identical three-dimensional objects presented in different orientations.	SV	3DSV	VBR	.571	.721
59	24	I can mentally visualize the three-dimensional geometric object formed by rotating a two-dimensional shape around an axis.	SV	3DSV	MR	.569	.626

41*	25*	I have difficulty perceiving the relative positions of objects after their locations are changed.	SR	3DSR	VBR	.791	.577
40*	26*	I have difficulty mentally visualizing the relative positions of shapes when their locations are changed.	SR	2DSR	VBR	.725	.647
61*	27*	I can classify objects by considering their directions and relative positions (in front of, behind, to the left, to the right, etc.)	SR	3DSR	VBR	.719	.560
26*	28*	I have difficulty accurately drawing the visual representation of a three-dimensional geometric object given its front, top, and side views.	SR	3DSR	VBR	.646	.643
43*	29*	I have difficulty mentally visualizing which shape appears on which face when viewing a cube with different shapes on each face from different directions.	SR	3DSR	VBR	.625	.674
47*	30*	When a geometric object composed of unit cubes is presented in different orientations, I have difficulty mentally visualizing how the cubes are arranged relative to one another.	SR	3DSR	VBR	.554	.597
49	31	I can relate structures encountered in daily life to geometric solids.	SO	2D⟺3D	PBR	.716	.491
54	32	I can draw a plan of the neighborhood in which I live.	SO	2D⟺3D	PBR	.705	.369
50	33	I can reach the same destination by using alternative routes.	SO	2D⟺3D	PBR	.679	.584
55	34	I can mentally visualize the three-dimensional form of a structure created by considering the direction and position information given in a building plan.	SO	2D⟺3D	PBR	.598	.569
48	35	I can assemble the parts of items requiring assembly by using the direction and position information provided in illustrated instructions.	SO	2D⟺3D	PBR	.559	.462

* Reverse-coded items; No1: Draft scale items; No2: Final scale items; F: Factor; SF: Sub-Factor; CP: Cognitive processes; FL: Factor loading; ITC: Item-total correlation; SA: Spatial ability; SV: Spatial visualization; SR: Spatial relations; SO: Spatial orientation; 2DSV: Two-dimensional spatial visualization; 3DSV: Three-dimensional spatial visualization; 2DSR: Two-dimensional spatial relations; 3DSR: Three-dimensional spatial relations; MI: Mental integration; MD: Mental decomposition; MR: Mental rotation; VBR: Viewpoint-Based Representation; PBR: Plan-Based Representation; $\alpha_{SA} = .960$; $\alpha_{SV} = .956$; $\alpha_{SR} = .872$; $\alpha_{SO} = .769$; $\alpha_{2DSV} = .859$; $\alpha_{3DSV} = .957$

Note: "Factor" refers to the psychometric structure of the scale, whereas "dimension" refers to the theoretical domains of spatial ability. In the SASEPS, the three main factors correspond to the theoretical dimensions of spatial visualization, spatial relations, and spatial orientation. Two-dimensional spatial visualization and three-dimensional spatial visualization are presented as sub-factors under the spatial visualization dimension. In addition, during the scale development process, particular attention was paid to the terminology used in order to ensure that the cognitive processes underlying the items were represented clearly and consistently. Accordingly, the terms shape, object, and solid were not used interchangeably or arbitrarily; rather, they were deliberately distinguished based on the specific spatial cognitive processes intended to be measured. In the scale, the term shape refers to two-dimensional spatial tasks involving planar geometric representations and operations such as decomposition, composition, pattern construction, comparison, and visualization. The term object, as commonly employed in the spatial cognition and psychology literature, refers to abstract three-dimensional geometric stimuli used to measure perceptual and dynamic spatial processes such as mental rotation, view recognition, and the perception of spatial relations. The objects used in this context do not correspond to real-world objects; rather, they consist of geometrically abstract, semantically neutral structures, such as Shepard–Metzler–type block figures. By contrast, the term solid is used in items that require the evaluation of nets, building and structural plans, closed–open transformations, and surface–edge relationships. The solids included in these items represent geometrically idealized three-dimensional structures with clearly defined structural properties and are primarily intended to assess whole–part relationships and processes of structural spatial analysis. This terminological distinction enables a more precise representation of the cognitive processes targeted by the scale items and strengthens the theoretical coherence of the scale with the broader literature on spatial cognition and geometry.

TABLE A2 Items removed from the scale.

The following items were removed from the scale because they loaded on a factor other than the intended three-factor structure.	
5*	I cannot mentally visualize a rotated version of a two-dimensional shape.
10	I can fold a piece of paper and create shapes.
15*	I have difficulty drawing the required three-dimensional objects.
19*	I cannot mentally visualize a rotated version of a three-dimensional objects.
28	I do not have difficulty decomposing a three-dimensional solid into parts and forming new three-dimensional solids.
30	I can determine how many cubes a given unit cube is in face-to-face contact with in structures composed of unit cubes
31	I can determine how many cubes are used in a structure composed of stacked or adjacent cubes.
32*	I have difficulty determining how many cubes are used in the top view of a structure composed of unit cubes.
33	I can determine how many cubes are required to complete a hollow structure composed of unit cubes.
34*	I have difficulty determining the number of cubes in structures composed of unit cubes.
35	I can calculate the number of unit cubes on the hidden surfaces of a solid composed of unit cubes.
38*	I can determine how many cubes are required to complete a hollow structure composed of unit cubes.
52	I can establish the relationship between shapes and solids.
The following items were removed from the scale because, although they did not disrupt the three-factor structure statistically, the factor they represented did not align theoretically with the factor determined through expert judgment.	
11*	I have difficulty creating shapes by folding paper.
12	After operations are applied to folded paper (e.g., punching holes or cutting), I can mentally visualize its unfolded form.
39	I can mentally visualize the position of a two-dimensional shape after it has been rotated.
56	I can draw the building plan of structure composed of unit cubes.
The following items were removed because their factor loadings were below .10.	
14	I can construct three-dimensional solids on paper.
16	I can recognize three-dimensional solids.
45*	I have difficulty mentally visualizing the position of a two-dimensional shape after it has been rotated.
46	I can construct the views of a structure composed of unit cubes from different orientations.
51*	I have difficulty finding a place I have never been to using navigation or directions.
53*	I cannot effectively use concepts of direction and orientation.
The following items were removed because their content validity ratios did not exceed .80.	
57*	I can relate structures encountered in daily life to geometric solids.
58	I can reach the same destination by using alternative routes.
60	I can give directional instructions (e.g., right, left, north, south) without thinking while giving directions.
62	I can draw a plan of the neighborhood in which I live.
63	I can construct a structure composed of unit cubes by using a given building plan.
64	I can draw the building plan of solids composed of unit cubes.

* Reverse-coded items

TABLE A3 Original Turkish version of the SASEPS

No	Madde	F	AF	BS
1	İki boyutlu şekillerden oluşan kurallı bir örüntüyü zihnimde görselleştirerek oluşturabilirim.	UG	2B UG	ZB
2	İki boyutlu bir şeklin hangi iki boyutlu şekillerin birleşmesiyle oluştuğunu bulabilirim.	UG	2B UG	ZA
3	İki boyutlu şekillerden oluşan bir örüntünün sonraki adımını zihnimde görselleştirerek çizebilirim.	UG	2B UG	ZB
4	Üç boyutlu geometrik nesnelerin iki boyutlu izdüşümlerini kâğıt üzerinde çizebilirim.	UG	2B UG	ZB
5*	İki boyutlu bir şekli parçalara ayırıp yeni iki boyutlu şekiller oluşturmakta zorlanırım.	UG	2B UG	ZA
6	Farklı pozisyonlarda verilen iki boyutlu şekiller arasından aynı olanları ayırt edebilirim.	UG	2B UG	G
7*	İstenilen iki boyutlu şekilleri çizerken zorlanırım.	UG	2B UG	G
8	İki boyutlu iki veya daha fazla şeklin birleşmesiyle oluşan şekilleri zihnimde canlandırabilirim.	UG	2B UG	ZB
9	Kapalı hali verilen üç boyutlu geometrik cisimlerin açılımını yapabilirim.	UG	3B UG	ZB
10	Üç boyutlu bir geometrik cismin farklı açılımlarını tanırım.	UG	3B UG	G
11	Üç boyutlu bir nesnenin döndürüldükten sonraki biçimini zihnimde oluşturabilirim.	UG	3B UG	ZD
12	Her bir yüzeyinde farklı şekiller bulunan bir küpün açılımını, yüzeylerindeki şekillerle birlikte yapabilirim.	UG	3B UG	ZB
13	Üç boyutlu nesnelerden oluşan bir örüntünün sonraki adımını zihnimde görselleştirerek çizebilirim.	UG	3B UG	ZB
14	Her bir yüzeyinde farklı şekiller bulunan küpü, farklı pozisyonlarda da tanıyabilirim.	UG	3B UG	G
15	Üç boyutlu geometrik nesnelerin farklı yönlerden iki boyutlu görünümlerini çizebilirim.	UG	3B UG	ZB
16	Üç boyutlu geometrik nesnelerin ara kesit yüzeylerini zihnimde canlandırabilirim.	UG	3B UG	ZB
17	Her bir yüzeyinde farklı şekiller bulunan ve açılımı verilen bir küpün, yüzeylerindeki şekilleri dikkate alarak kapalı hâlini oluşturabilirim.	UG	3B UG	ZB
18	Açılımı verilen üç boyutlu geometrik cisimlerin kapalı halini oluşturabilirim.	UG	3B UG	ZB
19	Üç boyutlu bir geometrik cismin yüzeylerinin hangi şekillerden oluştuğunu bulabilirim.	UG	3B UG	ZA
20	Farklı yönlerden iki boyutlu görünümleri verilen üç boyutlu geometrik nesnelerin görselini çizebilirim.	UG	3B UG	ZB
21	Üç boyutlu nesnelerden oluşan kurallı bir örüntüyü zihnimde görselleştirerek oluşturabilirim.	UG	3B UG	ZB
22	Üç boyutlu iki veya daha fazla geometrik nesnelerin birleşmesiyle oluşan geometrik nesneleri zihnimde oluşturabilirim.	UG	3B UG	ZB
23	Farklı pozisyonlarda verilen üç boyutlu nesneler arasından aynı olanları ayırt edebilirim.	UG	3B UG	G
24	İki boyutlu bir şeklin bir eksen etrafında döndürülmesiyle oluşan üç boyutlu geometrik nesnenin görselini zihnimde canlandırabilirim.	UG	3B UG	ZD
25*	Nesnelerin yeri değiştirildikten sonra birbirlerine göre olan konumunu algılamada zorlanırım.	Uİ	3B Uİ	G
26*	Şekillerin yerleri değiştirildiğinde, birbirlerine göre konumlarını zihnimde canlandırmakta zorlanırım.	Uİ	2B Uİ	G
27*	Nesneleri, birbirlerine göre yön ve konumlarını dikkate alarak sınıflandırabilirim (öndekiler, arkadakiler, soldakiler, sağdakiler…)	Uİ	3B Uİ	G
28*	Önden, üstten ve soldan/sağdan görünümü verilen üç boyutlu bir geometrik nesnenin görselini doğru biçimde çizmeye çalışırken zorlanırım.	Uİ	3B Uİ	G
29*	Her bir yüzeyinde farklı şekiller bulunan bir küpün farklı yönlerden görünümünde hangi şeklin hangi yüzeyde olacağını zihnimde canlandırmakta zorlanırım.	Uİ	3B Uİ	G
30*	Birim küplerden oluşan bir geometrik nesnenin görünümü farklı pozisyonlarda verildiğinde, küplerin birbirlerine göre nasıl dizildiğini zihnimde canlandırmakta zorlanırım.	Uİ	3B Uİ	G
31	Günlük hayatta karşılaştığım yapıları geometrik cisimlere benzetebilirim.	UY	2 B⟺3B	P
32	Yaşadığım mahallenin planını çizebilirim.	UY	2 B⟺3B	P
33	Alternatif yollar kullanarak aynı yere ulaşabilirim.	UY	2 B⟺3B	P
34	Yapı planında verilen yön ve konum bilgilerini dikkate alarak oluşturulan yapının üç boyutlu halini zihnimde canlandırabilirim.	UY	2 B⟺3B	P
35	Resimli talimatlardaki yön ve konum bilgilerini kullanarak montaj gerektiren eşyaların parçalarını birleştirebilirim.	UY	2 B⟺3B	P

* Ters maddeler; F: Faktör; AF: Alt faktör; BS: Bilişsel süreç; UY: Uzamsal yetenek; UG: Uzamsal görselleştirme; Uİ: Uzamsal ilişkiler; UY: Uzamsal yönelim; 2BUG: 2 boyutlu uzamsal görselleştirme; 3BUG: 3 boyutlu uzamsal görselleştirme; 2BUİ: 2 boyutlu uzamsal ilişkiler; 3BUİ: 3 boyutlu uzamsal ilişkiler ZB: Zihinde bütünleme; ZA: Zihinde ayrıştırma; ZD: Zihinde döndürme; G: Görünüm temelli temsil; P: Plan temelli temsil; $\alpha_{\text{UY}} = .960$; $\alpha_{\text{UG}} = .956$; $\alpha_{\text{Uİ}} = .872$; $\alpha_{\text{UY}} = .769$; $\alpha_{\text{2BUG}} = .859$; $\alpha_{\text{3BUG}} = .957$

APPENDIX 2 Sample item from the revised PSVT:R

Below is a sample item from the revised PSVT:R, as revised by Yoon (2011). This example is provided to illustrate the item format, operational procedure, and level of difficulty of the test. In the present study, a 30-item version of the test developed by Yoon (2011), consisting of items different from but similar in structure to the sample item, was used.

DIRECTIONS

This test consists of 30 questions designed to see how well you can visualize the rotation of three-dimensional objects. Shown below is an example of the type of question included in the second section.

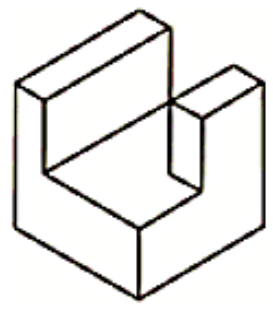

IS ROTATED TO

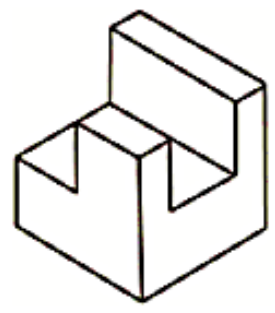

AS

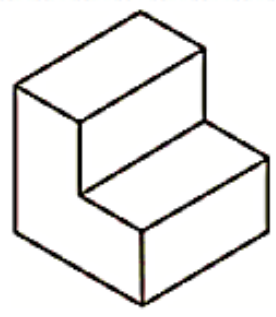

IS ROTATED TO

A B C D E

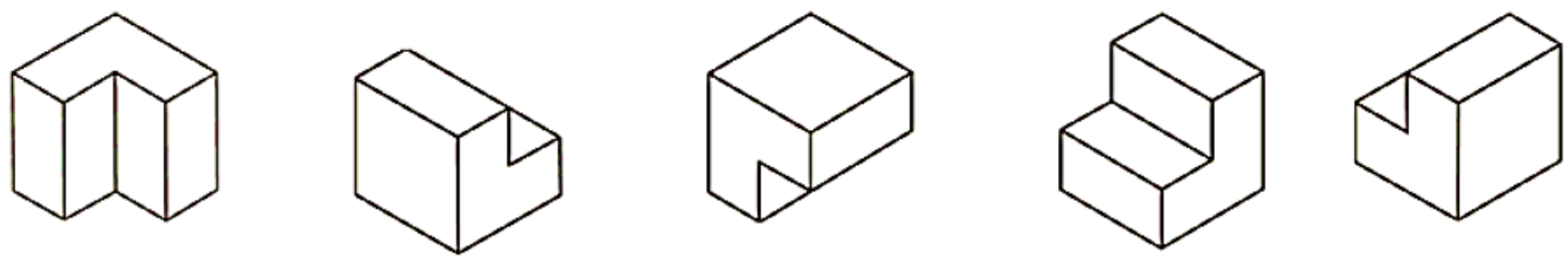

You are to:

1. study how the object in the top line of the question is rotated;
2. picture in your mind what the object shown in the middle line or the question looks like when rotated in exactly the same manner;
3. select from among the five drawings (A, B, C, D, or E) given in the bottom line of the question the one that looks like the object rotated in the correct position.

What is the correct answer to the example shown above?
